# Cardiovascular System Under Simulated Weightlessness: Head-Down Bed Rest vs. Dry Immersion

**DOI:** 10.3389/fphys.2020.00395

**Published:** 2020-05-19

**Authors:** Liubov Amirova, Nastassia Navasiolava, Ilya Rukavishvikov, Guillemette Gauquelin-Koch, Claude Gharib, Inessa Kozlovskaya, Marc-Antoine Custaud, Elena Tomilovskaya

**Affiliations:** ^1^Laboratory of Gravitational Physiology of the Sensorimotor System, Institute of Biomedical Problems, Russian Academy of Sciences, Moscow, Russia; ^2^Laboratoire MITOVASC, UMR Institut National de la Santé et de la Recherche Médicale 1083, Centre National de la Recherche Scientifique 6015, Université d’Angers, Angers, France; ^3^Centre National d’Etudes Spatiales, Paris, France; ^4^Institut NeuroMyogène, Université Claude Bernard Lyon 1, Lyon, France; ^5^Centre de Recherche Clinique, Centre Hospitalier Universitaire d’Angers, Angers, France

**Keywords:** support unloading, lower body negative pressure, water balance, orthostatic tolerance, autonomous regulation, microgravity models

## Abstract

**Background:**

The most applicable human models of weightlessness are −6° head-down bed rest (HDBR) and head-out dry immersion (DI). A detailed experimental comparison of cardiovascular responses in both models has not yet been carried out, in spite of numerous studies having been performed in each of the models separately.

**Objectives:**

We compared changes in central hemodynamics, autonomic regulation, plasma volume, and water balance induced by −6° HDBR and DI.

**Methods:**

Eleven subjects participated in a 21-day HDBR and 12 subjects in a 3-day DI. During exposure, measurements of the water balance, blood pressure, and heart rate were performed daily. Plasma volume evolution was assessed by the Dill–Costill method. In order to assess orthostatic tolerance time (OTT), central hemodynamic responses to orthostatic stimuli, and autonomous regulation, the 80° lower body negative pressure–tilt test was conducted before and right after both exposures.

**Results:**

For most of the studied parameters, the changes were co-directional, although they differed in their extent. The changes in systolic blood pressure and total peripheral resistance after HDBR were more pronounced than those after DI. The OTT was decreased in both groups: to 14.2 ± 3.1 min (vs. 27.9 ± 2.5 min before exposure) in the group of 21-day HDBR and to 8.7 ± 2.1 min (vs. 27.7 ± 1.2 min before exposure) in the group of 3-day DI.

**Conclusions:**

In general, cardiovascular changes during the 21-day HDBR and 3-day DI were co-directional. In some cases, changes in the parameters after 3-day DI exceeded changes after the 21-day HDBR, while in other cases the opposite was true. Significantly stronger effects of DI on cardiovascular function may be due to hypovolemia and support unloading (supportlessness).

## Introduction

Piloted space flights have been carried out for more than 50 years, yet the problem of cardiovascular deconditioning following microgravity exposure still exists and the problem of orthostatic stability disorders remains relevant. On the other hand, the study of the orthostatic instability mechanisms in astronauts may be complicated due to the small “*n*” and biased due to the obligatory use of onboard countermeasures. An alternative approach is the application of ground models to reproduce the major effects of microgravity in the human body, with the possibility of applying complex techniques at any time of exposure. The most applicable human models are head-down bed rest (HDBR) and dry immersion (DI; Shulzhenko and Vill-Villiams, 1976; At’kov and Bednenko, 1987; Grigor’ev et al., 2004; Pavy-Le Traon et al., 2007; Navasiolava et al., 2011a; Tomilovskaya et al., 2019). Being similar in their effects on the human body, these models, however, differ in their specifics and acting factors. The HDBR, as the name implies, implicates a long (from several weeks to a year) stay in the supine position. In our study, the position was anti-orthostatic (the head was tilted down by −6° to the horizontal). Thus, body liquids and the supporting surface are redistributed. The immersion is called “dry” because a waterproof film separates the subject from the water (unlike “wet” immersion, where the skin of a human or animal is in direct contact with the water). Due to the almost absolutely uniform weight distribution, the subject is affected by full support unloading. However, there are very few works comparing these models and their effects on the human body (Navasiolava et al., 2011a; Watenpaugh, 2016; Tomilovskaya et al., 2019). At the same time, a large number of original articles are devoted to the study of various systems in HDBR and DI. Thus, it makes sense to compare the effects of −6° HDBR and DI on human physiological systems, in particular the cardiovascular system.

In space, weightlessness immediately induces an upward fluid shift with the puffy face/chicken leg syndrome (Thornton and Hoffler, 1977). The onboard infrared photographs of the Skylab 4 crew members showed relatively empty lower limb veins, while the head veins were always fully filled and expanded (Gibson, 1977). The fluid shift leads to an increased venous return to the right heart. Receptors located in this zone give the signals about hypervolemia and initiate a decrease in the circulating plasma volume, mainly due to a decrease in fluid intake. As a consequence, the body fluid balance may be negative on the first day of exposure, after which a new equilibrium is established. Moreover, an increase in transcapillary filtration to the interstitial space contributes to a reduction in plasma volume (Watenpaugh et al., 2001).

In microgravity, the upward fluid shift initiates all subsequent changes in the cardiovascular system, including changes in the arterial and venous hemodynamics as well as in the vascular tone (Pestov and Gerathewohl, 1975; Gazenko, 1984; Kotovskaya and Fomina, 2013; Norsk et al., 2015). First of all, there is an increase in the stroke volume (SV; Buckey et al., 1996; Videbaek and Norsk, 1997), which leads to an increase in cardiac output by 18–26% because the heart rate is unchanged or decreased (Prisk et al., 1993; Fritsch-Yelle et al., 1996; Shykoff et al., 1996; Norsk et al., 2006, 2015).

While the central blood volume increases in weightlessness, the blood pressure either does not change or slightly decreases (Fritsch-Yelle et al., 1996; Shykoff et al., 1996; Norsk et al., 2006). During short-term space missions, a decrease in diastolic arterial pressure by 5 mmHg within the initial 2 weeks of spaceflight was reported, although there were no changes in systolic or mean arterial pressure. Therefore, because the cardiac output increased, the mean arterial pressure remained unchanged by the dilation of the arterial resistance vessels, inducing a decrease in systemic vascular resistance (Fritsch-Yelle et al., 1996; Shykoff et al., 1996; Norsk et al., 2006, 2015).

These changes are adaptive and normal for microgravity, but, upon returning to Earth, cardiovascular deconditioning can threaten the health of astronauts. Upon landing, a reverse fluid shift to the lower body occurs. Together with a reduced blood volume, this may compromise the adequate brain perfusion. The loss of muscle and vascular tone contributes to blood sequestration in the lower body. The prolonged absence of orthostatic stimuli during the spaceflight also leads to autonomic dysfunction and the inability to adequately respond to gravitational stimulus. Thus, without proper countermeasures, the astronaut may experience pre-syncope or syncope when upright (Martin and Meck, 2004).

Cardiovascular deconditioning is also characteristic of HDBR and DI, differing, however, in details. Head-down bed rest is the most popular model of microgravity since it provides a fairly accurate reproduction of most of the physiological effects of weightlessness due to immobilization, inactivity, and limitation of gravitational stimuli, such as posture and direction change (Fortney et al., 1996; Hargens and Vico, 2016; Watenpaugh, 2016; Klassen et al., 2018; Mulavara et al., 2018). Various angles of head-down tilt (usually −6°) can be used, contributing to a headward fluid shift (Greenleaf, 1984; At’kov and Bednenko, 1987; Grigor’ev et al., 2004). This thoraco-cephalic fluid shift and an increased venous blood flow to the right atrium together lead to changes in the secretion of vasopressin and aldosterone (Knight et al., 2009a, b). This results in a decrease in water reabsorption, an increase in sodium excretion by the kidneys, an increase in diuresis, and a decrease in plasma volume. It has been found that even an 8-h HDBR already causes an increase in blood supply to the head and chest by 6–9% compared with the initial horizontal position (Osadchii et al., 1997). In an experiment with exposure to a 7-day HDBR, the blood supply to the upper torso on the second and the seventh day has been shown to increase by 11 and 23%, respectively (Lobachik et al., 1991). A longer stay in HDBR is accompanied by the development of compensatory-adaptive reactions. Changes in plasma volume occur rather quickly and, after 6.5 h, reach a level of −9.2%. Despite the differences in the methods used, in general, the authors indicate a decrease in plasma volume by 6–15% with an HDBR duration from several days to a month and a half (Maillet et al., 1994; Sigaudo et al., 1996; Johansen et al., 1997; Blanc et al., 1998; Custaud et al., 2002). As in spaceflight, cardiovascular deconditioning characterized by orthostatic intolerance and reduced exercise capacity is observed at the end of bed rest (Pavy-Le Traon et al., 2007; Barbic et al., 2019).

The advantage of DI compared to the more widely known HDBR is support unloading (“supportlessness”), a state similar to weightlessness, with water hydrostatic pressure distributed equally over the body surface. The absence of support gradient provides conditions similar to a complete lack of support (Grigor’ev et al., 2004; Navasiolava et al., 2011a). Dry immersion promotes rapid gravitational deconditioning, which, for some systems (e.g., for the neuromuscular system), exceeds the deconditioning induced by spaceflight itself (Navasiolava et al., 2011a; Tomilovskaya et al., 2019). There is also evidence that DI has a more powerful effect on the cardiovascular system than does −8° HDBR (Krupina et al., 1982). Dry immersion, as well as HDBR, is accompanied by central hypervolemia, inducing an increase in cardiac dimension with heart stretching. For this reason, plasma volume decreases by approximately 15% within the first day of DI (Leach Huntoon et al., 1998) and remains stable thereafter (Gogolev et al., 1980; Larina et al., 2008; Nesterovskaia et al., 2008; Pakharukova et al., 2009; Navasiolava et al., 2011b). Decreased plasma volume is associated with diuresis and natriuresis (Epstein, 1992). The absence of changes in the levels of renin or aldosterone on days 3 and 7 is the evidence that the major redistribution of fluids is completed by that time and the water–electrolyte balance is stabilized.

Changes in blood supply to the vessels are followed by changes in central hemodynamics. The immediate effects of immersion in the first hours are an increase in SV and cardiac output, as well as a decrease in heart rate, blood pressure, and total peripheral vascular resistance (Modak and Banerjee, 2004; Bart et al., 2007; Ayme et al., 2014). An increase in cardiac output in the first hours of immersion is assumed to be associated with the redistribution of blood to the upper half of the body, while its decrease after 1 day is due to a decrease in the central blood volume as a result of the initiation of Parin and Henry–Gower reflexes. The parameters of blood pressure do not undergo significant changes. There is only a slight decrease in systolic blood pressure by 5–10 mmHg under immersion.

The idea of comparing the two models is not new. However, a detailed experimental comparison of the cardiovascular responses in both models has not yet been carried out, in spite of numerous studies having been performed using each of the models separately. Our analysis of the literature data allows us to suggest that the effect of DI may be stronger than that of HDBR. Therefore, we decided to test the hypothesis that the effects of 21-day −6° HDBR and 3-day DI for the cardiovascular system are comparable and to evaluate the optimal protocol (i.e., the optimal duration), which may be important for future studies. The aim of this work was to compare changes in the central hemodynamics, autonomic regulation, plasma volume, and water balance induced by the exposure to either −6° HDBR or DI.

## Materials and Methods

### Study Population

We analyzed raw data from two different experiments with participation of healthy European male volunteers: 21-day −6° HDBR (*n* = 11) and 3-day DI (*n* = 12). A comparison of these experiments was not part of the original study design. However, since the experimental protocols were identical, conducted and processed by the same team of authors, and both exposures are the model of microgravity physiological effects, we considered that comparing these data is reasonable. Both studies were performed at the MEDES Space Clinic (Toulouse, France) and conformed to the standards set by the Declaration of Helsinki. All subjects were informed about the experimental procedures and gave their written consent.

The first experiment analyzed in our paper was taken from the Medium duration Nutrition and vibration eXercise (MNX) Bed-Rest Study conducted from November 6, 2012 to December 20, 2013. This study was organized as three 21-day HDBR sessions (“Pure exposure,” “Exercise & vibration,” or “Exercise & vibration plus nutrition,” in a random order) separated by a 3-month washout. Moreover, all three sessions before HDBR did not significantly differ by hemodynamic parameters during the 80° tilt test from each other. The same volunteers participated in all three sessions. In this paper, we present data only from the “Pure exposure” session, referred to in the text as “HDBR exposure.” Twelve volunteers were recruited, but one dropped out of the experiment; thus, the data were obtained from 11 subjects. The MNX Bed-Rest Study was approved by the local Ethics Committee (CPP Sud-Ouest Outre-Mer I) and the French Health Authorities (no. ID RCB: 2012-A00337-36).

The second experiment was a 3-day head-out DI study conducted from January 13, 2015 to February 19, 2015. This study was organized as a single session. Twelve volunteers were recruited; all of them completed the session. The study was approved by the local Ethics Committee (CPP Sud-Ouest Outre-Mer I, France) and the French Health Authorities (no. ID RCB: 2014-A 00904-43).

Different volunteers participated in the 21-day −6° HDBR or the 3-day DI. Anthropometric data for the two groups were not significantly different (unpaired *t*-test with Welch’s correction, *p* < 0.05; Table 1).

**TABLE 1:** Comparative data of two groups.

Experimental groups	−6° head-down bed rest	Dry immersion	Significant differences (unpaired *t* test with Welch’s correction)
Duration (days)	21	3	–
Number of subjects, *n*	11	12	–
Age (years)	34 ± 2	32 ± 1	ns (*p* = 0.24, *t* = 1.23, *df* = 16.0)
Height (cm)	176 ± 2	178 ± 2	ns (*p* = 0.55, *t* = 0.61, *df* = 21.0)
Weight (kg)	70 ± 2	75 ± 2	ns (*p* = 0.17, *t* = 1.42, *df* = 19.8)
BMI (kg/m^2^)	22.4 ± 0.5	23.6 ± 0.4	ns (*p* = 0.13, *t* = 1.58, *df* = 20.1)

*Data are the mean ± SEM.*

### Study Protocol

In this study, the standard HDBR protocol was used. According to the experimental conditions, the subjects were lying for 21 days on the bed with a −6° inclination in the head direction. During the bed rest, the subjects continuously maintained a head-down position with their back or one shoulder and buttocks in contact with the bed. During HDBR, the subjects were not allowed to sit or to stand up. Moreover, they were allowed to use a pillow. All measurements and hygiene procedures were carried out in a horizontal position. The room temperature was set at 23–25°C.

Head-out DI lasted 3 days. Throughout the exposure, the subjects were in a bath filled with tap water and a waterproof film separated them from the water. The large surface area of the film allows the subject to easily be in the depth of the water and does not constrain his limbs. The size of the bath is designed in such a way that the subject does not touch its walls when immersed. The subjects were allowed to be immersed up to the armpits. The water temperature for DI was continuously maintained at 32.5–33.5°C (thermoneutral). During the immersion, the subjects remained continuously immersed, except for short out-of-bath periods for hygiene, weighing, and some specific measurements, when the subjects were maintained in the −6° head-down position. The total out-of-bath supine time within the 72 h of immersion was 4.7 ± 0.16 h (mean ± SEM).

**FIGURE 1:**
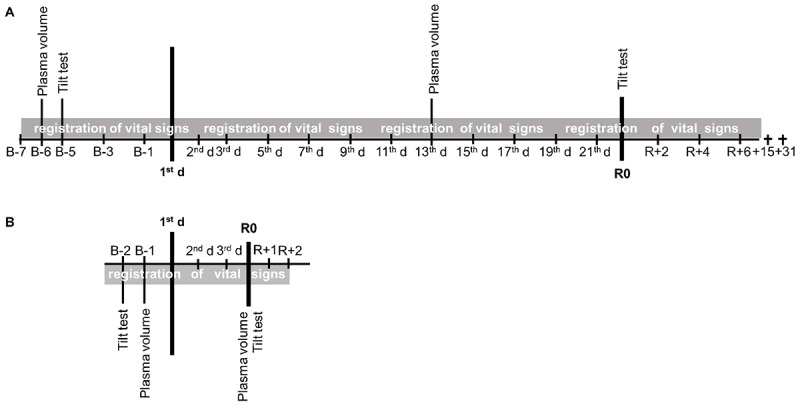
The protocols. **(A)** 21-day head-down bed rest (HDBR). **(B)** 3-day dry immersion (DI).

During both types of exposure, the subjects were under 24-h video monitoring. The beginning and end of both simulations occurred at 09:00 h. The light-off period was set at 23:00–07:00 h. Before, during, and after exposures, water intake was *ad libitum*. The diet was the same for all participants; it was standardized according to body weight in energy and nutrients based on WHO recommendations. The experimental protocols lasted 60 days (36 days in the facility) for the 21-day HDBR and 8 days for the 3-day DI. Comparative data on exposure times are presented in Figure 1.

### Measurements

#### Diuresis, Water Intake, and Partial Water Balance

Water intake and diuresis were measured daily beginning 7 days before HDBR and ending 6 days after it, as well as beginning 3 days before DI and ending 1 day after it. Water balance was calculated as the difference between the consumed water and urine volume. Water in exhaled air and sweating were not registered.

#### Plasma Volume Evolution

Blood sampling for hemoglobin (Hb) and hematocrit (Hct) was performed in the morning before breakfast at before exposure, as well as on the 13th day of HDBR and after 3 days of DI. The percent change in plasma volume vs. before exposure was calculated using the Dill and Costill formula (Dill and Costill, 1974): DPV(%) = 100 × [HbB (1 − 0.01 Hcti)]/[Hbi (1 − 0.01 HctB)] − 100, where HbB and HctB are the initial values and Hbi and Hcti are those during exposure.

#### Daily Blood Pressure and Heart Rate Measurement

Brachial blood pressure and heart rate were measured twice a day (at 7 a.m. and 7 p.m.) throughout the stay at the MEDES facility.

#### Lower Body Negative Pressure–Tilt Test

The tilt test combined with lower body negative pressure (LBNP) was performed at before exposures and at R0 immediately following simulation (first rising after HDBR and DI). Finger blood pressure (Nexfin, Bmeye, United States) and ECG (Biopac, MP150, United States) were continuously recorded in the supine position for 5 min, then in the 80° tilt position for 15 min, and then during LBNP steps of −10 mmHg every 3 min. The test was considered to be accomplished at −80-mmHg LBNP step for HDBR and at −60-mmHg step for DI. Orthostatic tolerance time (OTT) was measured as the entire verticalization period in accordance with the standard procedure described by Protheroe et al. (2013). The test was stopped earlier upon appearance of pre-syncopal signs, request to stop, systolic blood pressure ≤80 mmHg, and heart rate (HR) <50 bpm or >170 bpm.

To assess hemodynamic and autonomic responses to the tilt test, we selected the last 3 min of baseline stable supine recordings. In the tilt period, the last 3 min of stable records (excluding pre-syncope symptoms) were assessed. Heart rate, blood pressure (systolic, diastolic), SV, and total peripheral resistance (TPR) were determined. Autonomic cardiac modulation was assessed *via* heart rate variability (HRV) markers—normalized low- (LF) and high-frequency (HF) spectrum power, sympathetic index (LF/HF), and spontaneous baroreflex sensitivity (SBRS)—as detailed in De Abreu et al. (2017).

### Statistical Analysis

The results are presented as the mean ± SEM. All statistical analyses were performed using the GraphPad Prism program (8.3.0). Firstly, we assessed the normal distribution of anthropometric parameters by the Anderson–Darling test in groups (*p* > 0.05; the distribution is normal). We compared them using the unpaired *t*-test with Welch’s correction to ensure the groups were not significantly different (Table 1). Then, since the groups appeared comparable by anthropometric parameters, we compared the groups by other parameters. Three factors were used in the study: time (before and after exposure), models (HDBR and DI), and tilt (supine and 80° tilt). For comparison of the partial water balance in −6° HDBR and DI, we used two-way repeated measures ANOVA (time × models). Plasma volume evolution was analyzed by ordinary two-way ANOVA (time × models). Daily blood pressure and heart rate measurements were not compared between models because of the different durations of exposures. When comparing within each model, one-way repeated measures ANOVA was applied. Orthostatic tolerance time was tested by ordinary two-way ANOVA (tilt × models). Three-way ANOVA (time × models × tilt) was used for the hemodynamic parameters and HRV tilt test data. Bonferroni *post hoc* test was applied for all comparisons, the values of which are given in the text when the differences are significant. The significance level was set at α = 0.05.

## Results

### Diuresis, Water Intake, and Partial Water Balance

**FIGURE 2:**
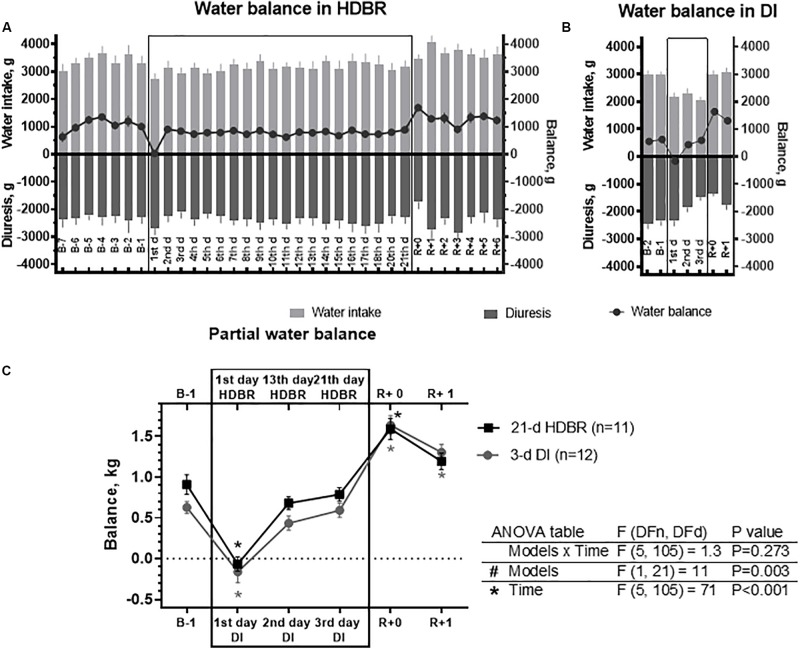
Changes in partial water balance in the 21-day head-down bed rest (HDBR) **(A)**, 3-day dry immersion (DI) **(B)**, and their comparison between both groups **(C)**. Data are the mean ± SEM. *p < 0.05 vs. before exposure.

Pre-bed rest water intake was 3.0–3.6 kg/day and diuresis was 2.3–2.7 kg/day; thus, water balance was positive and consisted of 0.6–1.3 kg/day (Figure 2A). Pre-immersion water intake was 3.0 kg/day, diuresis was 2.3–2.4 kg/day, and water balance consisted of 0.5–0.6 kg/day (Figure 2B).

On the first day of HDBR, there were a decrease in water intake and an increase in diuresis, which led to a nearly zero water balance (*p* < 0.001). However, on the second day of HDBR, water balance was positive and stabilized on a new level. On the first day of exposure to DI, a negative water balance was recorded (*p* < 0.001), the values of which exceeded those in the HDBR (Figure 2C). On the second and third days of immersion, water balance was stabilized on a positive level by reducing the water intake and diuresis (Figure 2B).

After the completion of either HDBR or DI, an increase in water balance was recorded (*p* < 0.001), which was due to an increase in the water intake and a decrease in diuresis. Starting from R + 1 day, water balance was not significantly different from the before exposure level. During the recovery period after DI, the water balance tended to restore; however, it did not reach the before exposure level.

We also compared the water balance at several time points: on the first, 13th, and 21st days for HDBR and on the first, second, and third days for DI (Figure 2C, upper and lower scales, respectively). As can be seen from the figure, the changes in water balance have a practically identical shape in both models. Although global two-way ANOVA was not significantly different in both time (*p* < 0.001) and model (*p* = 0.003) factors, the Bonferroni multiple comparisons *post hoc* test did not reveal differences in the latter.

It is worth noting that the total body mass of subjects progressively decreased during the experiments. The body mass loss was −3.4 kg (*p* < 0.001) on the 21st day of the −6° HDBR and was −1.4 kg (*p* < 0.001) on the third day of DI. Detailed data on the change in the body mass of subjects and the ratio of the partial water balance to it are given in the Supplementary Material.

### Plasma Volume Evolution

**FIGURE 3:**
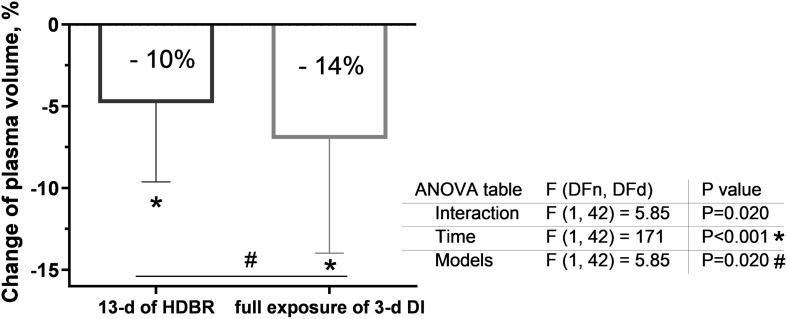
Changes in plasma volume on the 13th day of head-down bed rest (HDBR) and at the end of the 3-day dry immersion (DI). Data are the mean ± SEM. *p < 0.05 vs. before exposure; ^#^*p* < 0.05 between groups.

Plasma volume significantly decreased during both exposures (*p* < 0.001; Figure 3). At the end of the 3-day DI, the decrease in plasma volume was significantly greater (*p* = 0.003) than that on the 13th day of HDBR (14 ± 2% after DI vs. 10 ± 6% during HDBR).

### Daily Blood Pressure and Heart Rate Measurements

The data on blood pressure and heart rate changes during the experiments are presented in Tables 2 and 3. During HDBR, blood pressure did not significantly change; the heart rate became slightly decreased. On the first (*p* < 0.01), second (*p* < 0.01), third (*p* < 0.01), sixth (*p* = 0.01), seventh (*p* < 0.01), eighth (*p* = 0.03), and 13th (*p* = 0.01) days of HDBR, the heart rate was significantly decreased, and on the first (*p* = 0.01) and second (*p* < 0.01) days of recovery it was significantly increased compared to the before exposure level (average of 7 days before HDBR).

**TABLE 2:** Blood pressure and heart rate before, during, and after the 21-day −6° head-down bed rest (HDBR) at 7 pm.

		Average	B-7	B-6	B-5	B-4	B-3	B-2	B-1	1st day	2nd day	3rd day	4th day	5th day	6th day	7th day	8th day	9th ayd	10th day
SBP (mmHg)	Mean	116.4	117.5	118.9	114.5	113.5	114.9	118.9	116.9	113.6	113.9	113.5	117.5	114.0	113.7	116.5	114.6	114.6	114.3
	SEM	2.4	3.6	3.4	4.0	2.5	3.5	2.3	3.4	2.6	3.0	3.4	3.8	3.8	3.8	2.8	2.5	3.8	3.1
*DBP* (mmHg)	Mean	65.3	66.5	65.0	65.9	61.7	64.6	67.0	66.5	64.5	65.0	62.8	65.2	64.8	63.9	66.5	66.0	66.5	64.8
	SEM	1.2	1.6	2.1	1.7	2.4	1.8	1.6	1.5	2.2	0.89	2.7	1.8	1.3	2.2	2.2	1.4	1.4	1.5
HR (bpm)	Mean	59.8	51.6	59.4	71.4	57.5	65.4	58.7	54.4	50.5*	50.4*	49.6*	51.6	50.5	50.9*	50.4*	52.4*	53.4	53.0
	SEM	3.4	3.4	3.9	4.8	3.5	4.7	3.6	3.6	**2.3**	**2.4**	**2.4**	2.2	2.5	**2.6**	**2.3**	**2.9**	2.7	1.8

		**11th day**	**12th day**	**13th day**	**14th day**	**15th day**	**16th day**	**17th day**	**18th day**	**19th day**	**20th day**	**21st day**	**R0**	**R + 1**	**R + 2**	**R + 3**	**R + 4**	**R + 5**	**R + 6**

*SBP* (mmHg)	Mean	116.2	115.1	113.1	114.3	114.8	113.8	113.1	115.8	113.7	116.3	114.2	119.2	116.7	114.2	119.2	114.5	114.5	115.2
	SEM	4.1	2.2	1.2	4.6	3.9	3.4	2.2	3.5	3.1	2.5	3.0	2.8	4.0	2.8	2.8	3.5	2.7	3.3
DBP (mmHg)	Mean	67.3	66.3	62.9	66.7	65.5	63.6	65.0	66.0	63.1	66.7	66.4	70.2	69.2	65.5	71.0	65.9	65.8	65.8
	SEM	1.5	2.0	2.4	1.5	1.9	1.9	2.2	2.0	2.4	1.7	2.9	2.8	2.2	1.5	1.6	2.8	1.4	1.8
HR (bpm)	Mean	52.2	52.6	**51.7^∗^**	51.5	55.5	51.8	54.1	55.4	60.3	56.9	57.1	**69.2^∗^**	**77.0^∗^**	60.9	66.7	67.9	62.6	61.7
	SEM	1.5	2.7	**2.4**	2.0	3.3	1.8	2.8	2.4	3.9	2.3	2.2	**4.1**	**3.4**	3.7	3.4	4.1	3.7	3.0

Data are the mean ± SEM. *B*-, days before exposure; *R*+, days of recovery period. *p < 0.05 vs. average for 7 days before HDBR are indicated in bold.

**TABLE 3:** Blood pressure and heart rate before, during, and after the 3-day dry immersion (DI) at 7 pm.

		Average	B-3	B-2	B-1	DI 1	DI 2	DI 3	R0	R + 1
SBP (mmHg)	Mean	125.1	128.7	123.9	122.7	115.8*	121.5	125.9	130.5	128.7
	SEM	2.6	2.5	3.7	2.4	**3.5**	2.9	3.0	2.9	2.4
DBP (mmHg)	Mean	68.3	68.2	66.8	69.8	62.3*	65.1	69.8	74.2*	74.0
	SEM	1.7	1.7	1.8	2.3	**1.9**	2.2	1.3	**1.9**	2.3
HR (bpm)	Mean	56.4	56.9	58.4	53.9	59.0	53.9	58.0	69.4*	58.3
	SEM	1.9	2.1	2.3	1.6	3.47	1.9	2.8	**2.6**	2.1

Values are the mean ± SEM. *B*-, days before exposure; *R* +, days of recovery period. *p < 0.05 vs. average for 3 days before DI are indicated in bold.

During DI, the first day was marked by decreases in systolic blood pressure (SBP; *p* = 0.027) and diastolic blood pressure (DBP; *p* < 0.01), whereas heart rate did not change. On the first day of recovery, diastolic blood pressure (*p* = 0.048) and heart rate (*p* < 0.01) significantly increased compared to the average of 3 days before DI.

### Hemodynamic and Autonomic Responses to the Tilt Test

**FIGURE 4:**
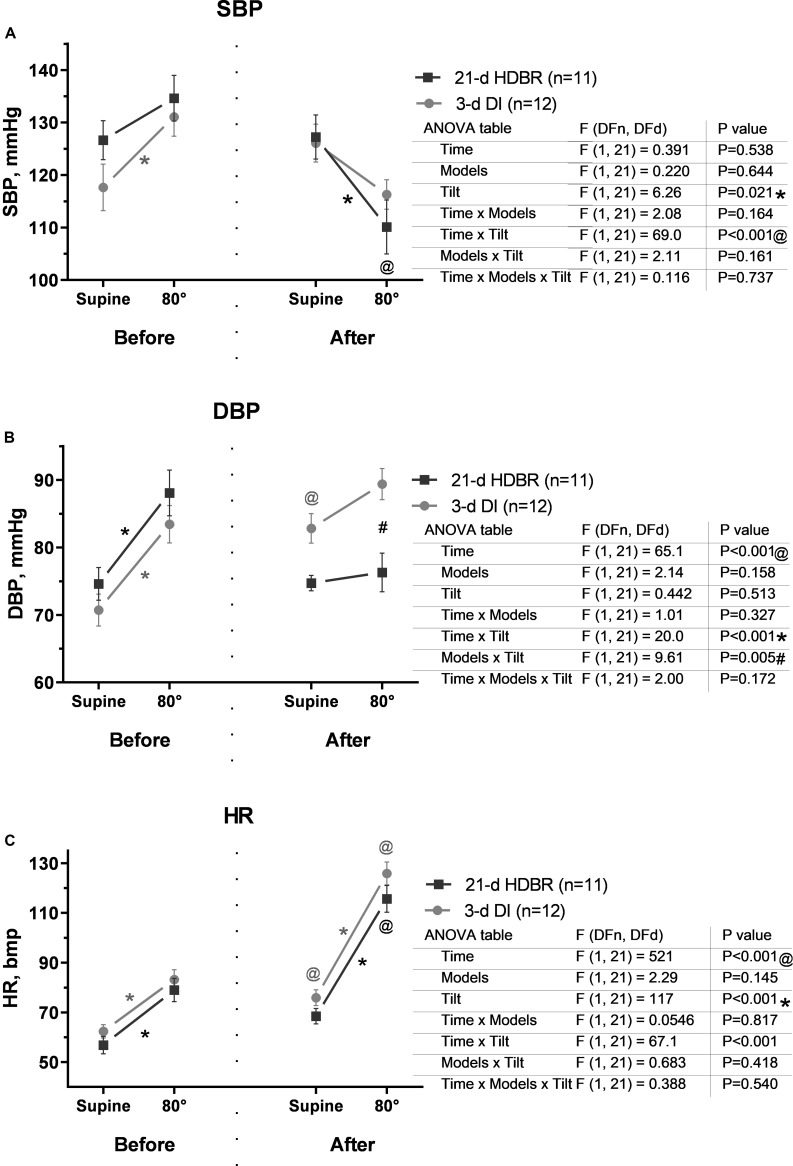
Changes in systolic blood pressure **(A)**, diastolic blood pressure **(B)**, and heart rate **(C)** in response to the 80° tilt test before and after the 21-day head-down bed rest (HDBR) and 3-day dry immersion (DI). Data are the mean ± SEM. ^#^*p* < 0.05 between groups; *p < 0.05, 80° vs. the supine position; ^@^*p* < 0.05, before vs. after exposure.

Before either HDBR or DI exposure, SBP during the tilt test increased in both groups vs. the supine position (*p* = 0.02 for DI; Figure 4A). After the 21-day HDBR and 3-day DI, the changes in SBP had the opposite characteristic: SBP dropped by ∼17 mmHg after HDBR (*p* = 0.002) and by ∼10 mmHg after DI exposures.

Diastolic blood pressure during the tilt test increased significantly vs. the supine position compared to that before HDBR and DI (*p* < 0.001; Figure 4B). After either the 21-day HDBR or 3-day DI, a DBP increase during table rotation was less pronounced than that before exposures, especially for HDBR. Diastolic blood pressure in the supine position was higher after DI compared to that before exposure (*p* = 0.046).

Before either HDBR or DI exposure, heart rate during the tilt test increased in both groups by ∼12 bpm vs. the supine position (*p* < 0.001; Figure 4C). After exposures, HR was higher by 11–13 bpm even at rest (*p* = 0.047 for DI). During the tilt test, HR significantly increased by 65–70% vs. the supine position (*p* < 0.001) and by 47–50% vs. before exposure (*p* < 0.001) in both groups.

**FIGURE 5:**
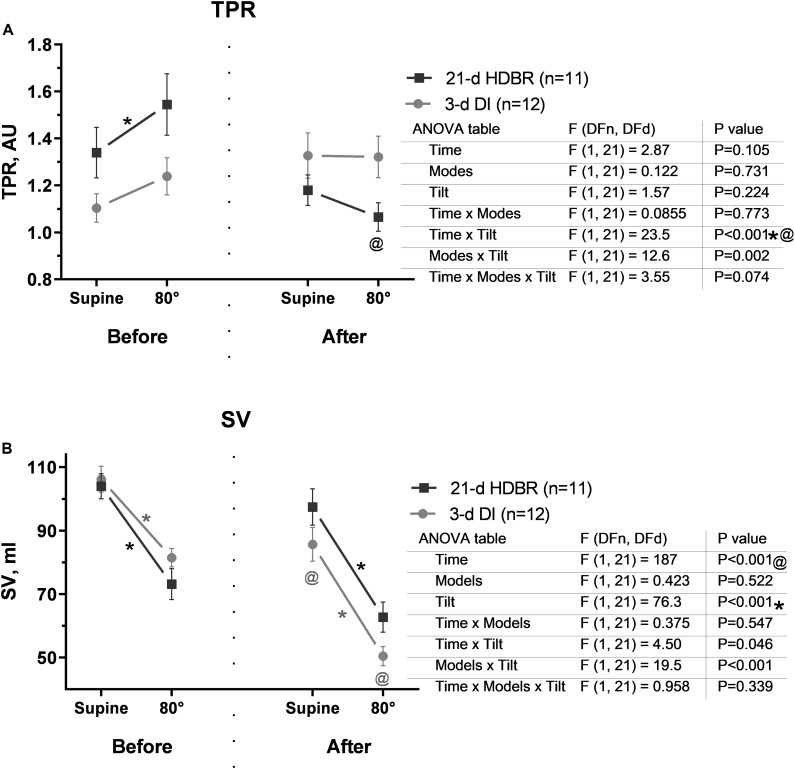
Changes in total peripheral resistance **(A)** and stroke volume **(B)** in response to the 80° tilt test before and after the 21-day head-down bed rest (HDBR) and 3-day dry immersion (DI). Data are the mean ± SEM. ^#^*p* < 0.05 between groups; *p < 0.05, 80° vs. the supine position; ^@^*p* < 0.05, before vs. after exposure.

Before both exposures, the TPR during the tilt test increased (*p* = 0.029 for HDBR; Figure 5A). After exposures, during the tilt test, the TPR had a slight tendency to increase in the DI group and to decrease in the HDBR group compared to those before exposures (*p* < 0.001).

Before either HDBR or DI exposure, SV during the tilt test decreased (*p* < 0.001; Figure 5B). After exposures, the SV decrease during the tilt test was more pronounced. However, after DI, these changes achieved significance compared to those before exposure (*p* < 0.001).

**FIGURE 6:**
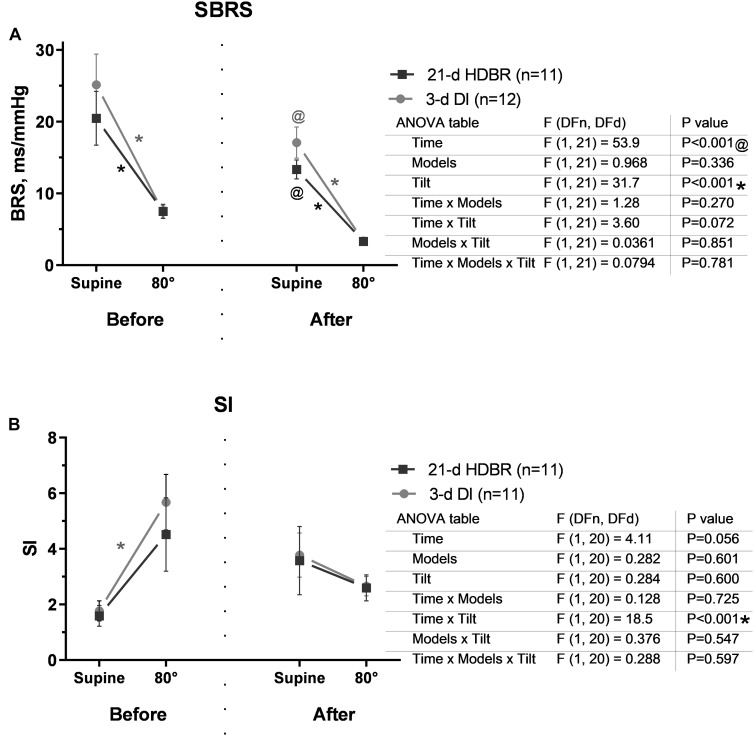
Changes in spontaneous baroreflex **(A)** and sympathetic index **(B)** in response to the 80° tilt test before and after the 21-day head-down bed rest (HDBR) and 3-day dry immersion (DI). Data are the mean ± SEM. ^#^*p* < 0.05 between groups; *p < 0.05, 80° vs. the supine position; ^@^*p* < 0.05, before vs. after exposure.

Spontaneous baroreflex sensitivity during the tilt test significantly dropped compared to that before exposures (*p* = 0.002 for HDBR and *p* < 0.001 for DI; Figure 6A). After HDBR and DI, supine SBRS was lower than the initial level (*p* = 0.026 for HDBR and *p* = 0.004 for DI), decreasing further in response to tilt (*p* = 0.047 for HDBR and *p* < 0.001 for DI). The changes in both groups were similar.

Before either HDBR or DI exposure, an increase in sympathetic index (SI) reflecting cardiac sympathetic activation was observed in response to orthostasis (*p* = 0.030 for DI). After the 21-day HDBR and 3-day DI, SI was slightly increased even at rest (Figure 6B) and had a tendency to increase during the tilt test.

### Orthostatic Tolerance

**FIGURE 7:**
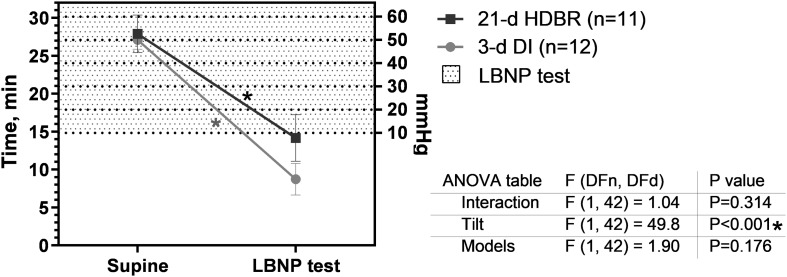
Orthostatic tolerance time before and after the 21-day head-down bed rest (HDBR) and 3-day dry immersion (DI). Data are the mean ± SEM. *p < 0.05 vs. before exposure.

The OTT is an integrative measure of the success of the strategy to provide the upright position of the body. Before both exposures, OTT consisted of 27–28 min, corresponding to ∼50 mmHg (Figure 7, right scale) of LBNP. After exposures, the OTT decreased in both groups (*p* < 0.001): to 14.2 ± 3.1 min after the 21-day HDBR and to 8.7 ± 2.1 min after the 3-day DI.

## Discussion

### Main Findings

The main finding is that cardiovascular changes induced by the 21-day −6° HDBR and 3-day DI are comparable, despite the sevenfold difference in the duration of exposures.

Both models reproduce the absence of physical loads. Thus, deep hypokinesia is reproduced in both models. The strict horizontal position (without daily raise) during the protocols of HDBR and DI has a detraining effect on the cardiovascular and other systems. However, the degree of reproduction of such an important factor as support unloading differs: in HDBR, support loads are redistributed from the soles to the surface of the back, buttocks, and the back surfaces of the legs; in DI, there is virtually no support due to buoyancy. We believe that support unloading is an important factor in the development of microgravitational deconditioning and that its increase leads to stronger effects in a short time.

#### Fluid Shift Influence

The parameter that differs between the −6° HDBR and DI models is the mechanism that provides the fluid shift. In HDBR, it is achieved by an anti-orthostatic position, which promotes fluid transition to the upper parts of the body. A number of authors have shown that the fluid shift processes during HDBR occur quite quickly. Water intake, diuresis, and plasma volume stabilized on a new level by the third to fourth day or earlier (Greenleaf, 1983; Meck et al., 2009; Navasiolava et al., 2011a). In our case, the water balance was established on the second day of HDBR. In the immersion, in contrast to HDBR, hydrostatic compression induces fluid centralization. According to a review article by E. Tomilovskaya et al., fluid shift occurs in DI faster than in HDBR (during the first day) (Tomilovskaya et al., 2019). Interestingly, in our study, the effect of the 3-day DI on water intake and diuresis was more pronounced (but not significantly) than in the 21-day −6° HDBR. In both cases, the fluid shift occurs, simulating the conditions of spaceflight.

In our study, there was a significant progressive decrease in the subject’s body mass. However, surprisingly, fluid loss did not play a dominant role in this. Before the exposures, all subjects lived in a hospital (for 7 days before HDBR and 3 days before DI) and received a standard regulated diet. We assume that the transition to a more proper and balanced nutrition could contribute to a weight loss in our study. Also, we suggest that body mass decrease in the last stages of HDBR could be associated with muscle loss, which is observed in other bed rest studies (Shenkman et al., 1997; Stuempfle and Drury, 2007; Dirks et al., 2016; Kramer et al., 2017; Shenkman and Kozlovskaya, 2019).

#### Central Hemodynamic Parameters

Despite the similarity of the cardiovascular changes, we observed a number of differences in the effects of the two models. After the completion of the 21-day HDBR and 3-day DI, resting tachycardia and orthostatic intolerance were detected, accompanied by a relative decrease in upright SBP, DBP, SV, and TPR and an increase in HR in response to tilt. Our results are in agreement with literature data (Buckey et al., 1996; Knight et al., 2009a) and indicate a significant cardiovascular deconditioning after both simulations. Interestingly, supine TPR and DBP had a tendency to increase following DI, but not HDBR. These parameters may suggest an increase in initial vascular tone, the important role of which was indicated by several authors (Convertino et al., 1999; Vinogradova et al., 2002). According to the Convertino hypothesis (Convertino et al., 1999), the diminished vasoconstrictive reserve may be the main mechanism of vasoconstrictor insufficiency in case of orthostatic intolerance. The maximal capacity of vasoconstriction is not altered under microgravity (Convertino et al., 1999), but hypovolemia may induce an increase in initial vasoconstriction and, thus, decrease the vasoconstrictive reserve (Convertino et al., 1999).

#### Autonomic Regulation of Cardiovascular Functions

Autonomic regulation is extremely important in maintaining blood pressure homeostasis during verticalization (Mano, 2005). In our studies, baroreflex sensitivity was reduced both at rest and during the tilt test after exposure, suggesting a reduced capacity of the baroreflex loop to regulate blood pressure (Tank et al., 1995). Both after the 21-day HDBR and 3-day DI, the SI failed to increase in the upright position, which is one of the signs of autonomic insufficiency.

#### Orthostatic Intolerance

The time of orthostatic stability, an integrative parameter of the cardiovascular state, demonstrates the efficacy of the strategy of vertical stance maintenance. In our study, the OTT after both exposures decreased without a significant difference between groups. However, the signs of orthostatic insufficiency observed after the 3-day DI tended to be more pronounced (8.7 min in DI vs. 14.2 min in HDBR), probably due to a more pronounced post-exposure hypovolemia and diminished vasoconstrictive reserve.

#### Evaluation of the Optimal Protocol

DI was seven times shorter than HDBR, yet we detected similar changes in the studied parameters, which suggest an accelerated cardiovascular impairment in DI compared to HDBR. However, the cardiovascular deconditioning appears rather quickly and then remains at a rather stable level; probably, comparing experiments of the same duration would show the same degree of cardiovascular deconditioning, which undoubtedly requires verification. Seventy-five percent of the 3-day DI subjects and 55% of the 21-day HDBR subjects were not able to complete the tilt test. This complies with literature data on HDBR of various durations: 5 out of 11 (45%), after 4 days (*p* = 0.15); four out of six (67%), after 14 days (*p* = 0.7); five out of nine (56%), after 28 or 30 days (*p* = 0.35); and four out of seven (57%), after 42 days of HDBR (*p* = 0.4) (Pavy-Le Traon et al., 2007).

It is interesting to note that, when applying two- or three-way ANOVA to analyze central hemodynamic parameters, HRV, and plasma volume, the interaction of such factors as time and tilt was identified, which may indicate that they are co-directional. The interaction of the model and tilt factors was found in DBP, TPR, and SV. However, possible interpretations should be made with caution.

### Study Limitation

The protocols of the 21-day HDBR and 3-day DI were followed independently of each other; therefore, their durations differed by seven times. However, despite the fact that the use of the same research methods in both models made it possible to compare the obtained data, it is worth reminding that it was not planned for as the original protocols, and this may introduce certain limitations. Still, it is of interest to make consistent experimental comparisons of protocols of the same duration [both short (3–5 days) and longer (several weeks)].

Increasing the sample size would also have a positive effect on the reliability of the results. However, a sample of 10–12 subjects is quite common in space biology studies.

## Conclusion

In general, cardiovascular changes during the 21-day −6° HDBR and head-out 3-day DI were co-directional. Frequently, changes after 3-day DI were equal to or exceeded changes after 21-day HDBR. Significantly stronger effects of DI on cardiovascular function can be caused not only by a more pronounced hypovolemia but also by support unloading (supportlessness). The support deafferentation plays a trigger role in the development of hypogravitational disorders. This was shown for the sensorimotor system (Grigor’ev et al., 2004; Kozlovskaya et al., 2007); however, for other systems, the role of support afferentation is under question. A decrease in postural muscle tone in response to a decrease in support afferentation may be responsible for the orthostatic impairment *via* a decrease in the efficiency of the muscle pump promoting venous return.

## Data Availability Statement

The datasets generated for this study are available on request to the corresponding author.

## Ethics Statement

MNX Bed-Rest study was approved by the local Ethic Committee (CPP Sud-Ouest Outre-Mer I) and French Health Authorities (NO ID RCB: 2012-A00337-36). Dry Immersion study was approved by the local Ethic Committee (CPP Sud-Ouest Outre-Mer I, France) and French Health Authorities (NO ID RCB: 2014-A 00904-43). The patients/participants provided their written informed consent to participate in this study.

## Author Contributions

LA conducted measurements, processing results and writing the manuscript. NN participated in critically revision of the manuscript. IR, GG-K and CG helped to organize the DI procedure in MEDES and participated in discussion of the results. IK, M-AC and ET participated in concepting of the idea and critical revision of the manuscript.

## Conflict of Interest

The authors declare that the research was conducted in the absence of any commercial or financial relationships that could be construed as a potential conflict of interest.

## Abbreviations

ANOVA: analysis of variance
B-: days before exposure
DBP: diastolic blood pressure
DI: dry immersion
ECG: electrocardiogram
HDBR: head down bed rest
HR: heart rate
LBNP: lower body negative pressure
OTT: orthostatic tolerance time
PV: plasma volume
R0: end-exposure day
R+: days of recovery period
SBP: systolic blood pressure
SEM: standard error of the mean
SV: stroke volume
TPR: total peripheral resistance
