# Supplementary material for: Cardiovascular System Under Simulated Weightlessness: Head-Down Bed Rest vs. Dry Immersion
Source: Front Physiol. 2020 May 19;11:395. doi: 10.3389/fphys.2020.00395 (PMC7248392; doi:10.3389/fphys.2020.00395)
Supplement: Supplementary file 1 [file Data_Sheet_1.PDF]

Supplementary Material

1 Supplementary data on the weight of subjects during 21-d -6° Head Down Bed Rest and 3-d Dry Immersion

Measurement of total body weight was carried out in the morning (7 am). The tables I and II show the average data of subjects for each day of experiments. Repeated measures one-way ANOVA with Bonferroni multiple comparisons test was carried out. Statistical significance was analyzed relative to average before exposures values.

Table I. Body mass of subjects before, during and after 21-day HDBR. Mean±SEM. *p < 0.05 vs. average for 7 days before HDBR

		average	***B -7***	***B -6***	***B -5***	***B -4***	***B -3***	***B -2***	***B -1***	***1st d***	***2nd d***	***3rd d***	***4th d***	***5th d***	***6th d***	***7th d***	***8th d***	***9th d***	***10th d***
Body mass, kg	Mean	71,3	71,9	71,6	71,2	71,3	71,3	71,1	70,9	70,9	**70,1***	**70,1***	**70,0***	**69,8***	**69,8***	**69,8***	**69,6***	**69,5***	**69,7***
	SEM	3,0	3,0	2,9	3,0	3,0	3,1	3,0	3,1	3,1	**3,0**	**3,0**	**3,0**	**3,1**	**3,0**	**3,1**	**3,1**	**3,1**	**3,0**

		11th d	***12th d***	***13th d***	***14th d***	***15th d***	***16th d***	***17th d***	***18th d***	***19th d***	***20th d***	***21th d***	***R 0***	***R+ 1***	***R+ 2***	***R+ 3***	***R+ 4***	***R+ 5***	***R+ 6***
Body mass, kg	Mean	**69,2***	**69,0***	**69,0***	**69,0***	**69,0***	**68,7***	**68,7***	**68,6***	**68,5***	**68,2***	**68,2***	**68,3***	**68,8***	**69,3***	**69,3***	**69,0***	**69,2***	**69,2***
	SEM	**3,1**	**3,1**	**3,1**	**3,1**	**3,1**	**3,1**	**3,1**	**3,1**	**3,1**	**3,2**	**3,2**	**3,2**	**3,2**	**3,2**	**3,3**	**3,2**	**3,2**	**3,2**

Table II. Body mass of subjects before, during and after 3-day DI. Mean±SEM. *p < 0.05 vs. average for 3 days before exposures)

		average	***B-3***	***B-2***	***B-1***	***DI 1***	***DI 2***	***DI 3***	***R0***	***R+1***
Body mass,	Mean	75,1	74,5	75,5	75,4	74,9	**73,8***	**73,7***	**73,7***	**74,2***
kg	SEM	2,1	2,1	2,1	2,1	2,1	**2,0**	**2,0**	**2,0**	**2,1**

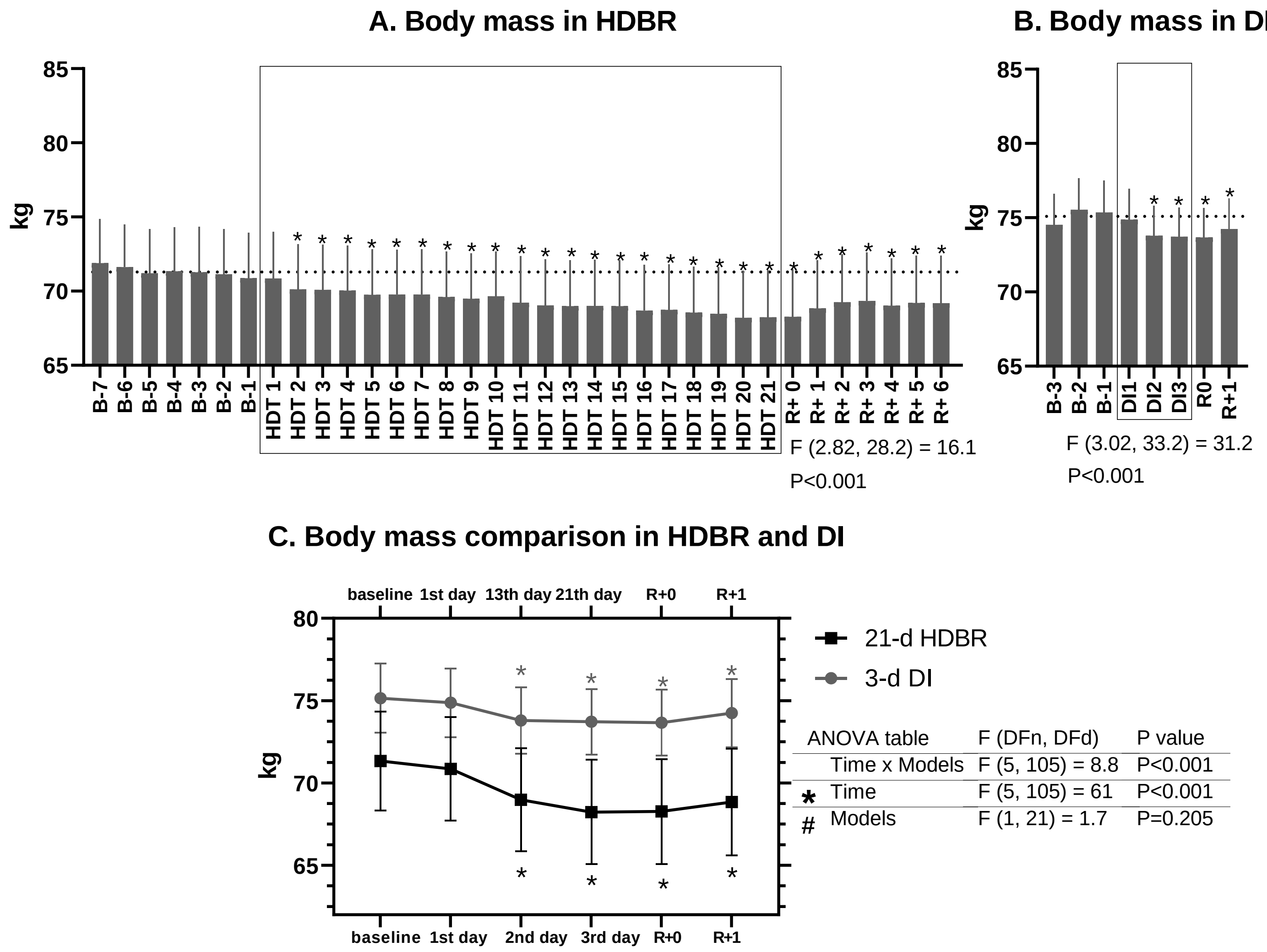

Figure I. Changes of body mass in 21-day HDBR (A), 3-day DI (B) and its comparison in both groups (C). Data are mean ± SEM. *p < 0.05 vs. before exposure.

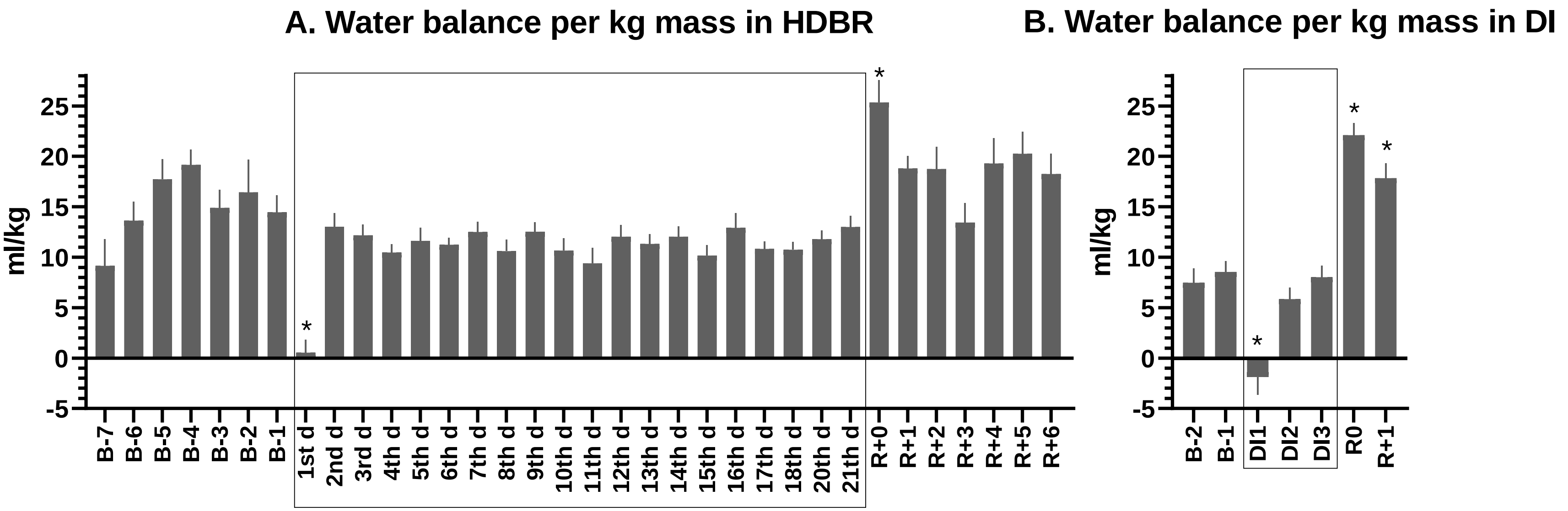

Figure II. Changes of water balance normalized to body mass in 21-day HDBR (A) and 3-day DI (B). Data are mean ± SEM. *$p < 0.05$ vs. before exposure (B-1).
